# Selective control of gait subtasks in robotic gait training: foot clearance support in stroke survivors with a powered exoskeleton

**DOI:** 10.1186/1743-0003-10-3

**Published:** 2013-01-21

**Authors:** Bram Koopman, Edwin HF van Asseldonk, Herman van der Kooij

**Affiliations:** 1 Institute for Biomedical Technology and Technical Medicine (MIRA), Department of Biomechanical Engineering, University of Twente, Enschede, The Netherlands; 2 Department of Biomechanical Engineering, Delft University of Technology, Delft, The Netherlands

**Keywords:** Robotic gait rehabilitation, Stroke, Reference trajectories, Virtual model control, Support of subtasks, Adaptive control, Impedance control, Reliance, Compensatory strategies, Visual feedback

## Abstract

**Background:**

Robot-aided gait training is an emerging clinical tool for gait rehabilitation of neurological patients. This paper deals with a novel method of offering gait assistance, using an impedance controlled exoskeleton (LOPES). The provided assistance is based on a recent finding that, in the control of walking, different modules can be discerned that are associated with different subtasks. In this study, a Virtual Model Controller (VMC) for supporting one of these subtasks, namely the foot clearance, is presented and evaluated.

**Methods:**

The developed VMC provides virtual support at the ankle, to increase foot clearance. Therefore, we first developed a new method to derive reference trajectories of the ankle position. These trajectories consist of splines between key events, which are dependent on walking speed and body height. Subsequently, the VMC was evaluated in twelve healthy subjects and six chronic stroke survivors. The impedance levels, of the support, were altered between trials to investigate whether the controller allowed gradual and selective support. Additionally, an adaptive algorithm was tested, that automatically shaped the amount of support to the subjects’ needs. Catch trials were introduced to determine whether the subjects tended to rely on the support. We also assessed the additional value of providing visual feedback.

**Results:**

With the VMC, the step height could be selectively and gradually influenced. The adaptive algorithm clearly shaped the support level to the specific needs of every stroke survivor. The provided support did not result in reliance on the support for both groups. All healthy subjects and most patients were able to utilize the visual feedback to increase their active participation.

**Conclusion:**

The presented approach can provide selective control on one of the essential subtasks of walking. This module is the first in a set of modules to control all subtasks. This enables the therapist to focus the support on the subtasks that are impaired, and leave the other subtasks up to the patient, encouraging him to participate more actively in the training. Additionally, the speed-dependent reference patterns provide the therapist with the tools to easily adapt the treadmill speed to the capabilities and progress of the patient.

## Background

Many patients with neurological injuries, like stroke or spinal cord injury (SCI), suffer from muscle weakness, loss of independent joint control, and spasticity, often resulting in gait disorders. To regain functional mobility, these patients require task-oriented, high-intensity, and repetitive training [1-3]. Robotic gait-training devices are increasingly being used to provide this kind of training. They can provide highly repetitive, more frequent, and intensive training sessions, while reducing the workload of the therapist, compared to more conventional forms of manual-assisted (and body-weight-supported) gait training. Additionally, the assessment of the progress of the patient becomes more objective with the integration of different sensory systems, which can record interaction forces and gait kinematics [4].

Despite the reduction in labor intensity, the therapeutic effect of the different types of gait trainers is inconsistent. Pohl, et al., and Mayr, et al., reported a significant improvement in gait ability in subacute stroke patients, compared to conventional physiotherapy [5,6]. Other studies found no significant difference between robotic support and manual treadmill training [7,8], or conventional physiotherapy [9], although robotic gait training did show improvements in gait symmetry [7,9]. Some results even indicate that manual treadmill training is superior to robotic assistance [10]. Recently, a large multicenter randomized clinical trial suggested that the diversity of conventional gait training elicits greater improvements in functional recovery than robotic-assisted gait training [11]. These contradicting results emphasize that robot-aided training needs to be further optimized to increase therapeutic outcome.

One of the most important factors that promotes therapeutic outcome is active participation. Active patient participation has been proven to be beneficial for motor learning in general [12-14] and is suggested to be important for rehabilitation of gait disorders [15]. The “first-generation” devices, like the Lokomat (Hocoma AG, Switzerland) or AutoAmbulator (HealthSouth, USA), were initially developed based on the approach of enforcing gait upon a patient by moving the legs through a prescribed gait pattern. This diminishes the need for the patients to actively contribute to the required motion. Moving the legs in a rigid fashion is known to reduce [16] and affect [17] voluntary muscle activity compared to manual assistance, possibly making the patient reliant on the support. Rigid trajectory control also limits the natural gait variability and the possibility to make small movement errors. These small errors have been suggested to promote motor learning in mice [18] as well as humans [19,20].

To encourage active participation, and allow natural gait variability, more and more robotic devices control the interaction forces by using impedance or admittance control algorithms [21-29]. They guide the leg by applying a force rather than imposing a trajectory. Impedance (or admittance) control can also make the robot’s behavior more flexible and adaptive to the patient’s capabilities, progress, and current participation. Depending on the impedance levels, small errors are still possible, promoting motor recovery. Patients might also increase their motivation, since additional effort by the patient is reflected in their gait pattern. Controllers based on this principle are referred to as “assist-as-needed” (AAN), “cooperative,” “adaptive,” or “interactive” controllers. In mice, these AAN algorithms have been shown to be more effective than position-controlled training [30].

Using impedance control instead of position control, however, introduces new challenges. First, low impedance levels increase the risk that the subject and robot start to walk out of phase. Consequently, the robot will resist, rather than support, the subject. Different algorithms have been proposed to avoid synchronization problems. To account for alterations in cadence, the reference pattern of the robotic controller can be accelerated or decelerated, based on the difference between the current gait phase of the subject and the state of the robot. This can be done continuously [21] or on a step-by-step basis [27].

Second, the impedance level needs to match the patient’s capabilities and progress, which can vary widely due to different levels of increased muscle tone, muscle weakness, or loss of coordinated control. This makes choosing the appropriate setting a priori a difficult process for the operator. In most applications, the amount of support is set by the operator on a trial-and-error basis. Setting the support levels too low can result in a dangerous situation, whereas too much assistance might reduce active participation of the patient. Roughly two strategies can be distinguished to automate the process of setting the support levels. The support levels can be adjusted based on increased patient effort (detected with force sensors) [24], or based on kinematic errors [27]. Emken, et al. [27], developed an error-based controller with a forgetting factor. The algorithm systematically reduces the impedance levels when kinematic errors are small, whereas it increases the impedance when the errors are large. When the subject (unconsciously) reduces his effort, he will experience no support. Only when the subject fails to commit to the reference pattern for a longer duration of time, the support will be increased. This should prevent the patient from becoming reliant on the support. In parallel, it allows normal gait variability by lowering the impedance levels, when possible. Others use a deadband or a non-linear stiffness to allow normal variability, without causing the robot to increase its assistive forces [25,29].

Third, even when the impedance levels are adaptive, the whole movement is still potentially supported. This implies that the patient receives support during gait phases where his performance decreases, making no distinction between the patient’s incapability, reliance, or fatigue. This also limits the possibility to focus the therapy on specific aspects of the walking pattern that require special attention.

Fourth, despite that impedance control does not rigidly impose a fixed reference pattern, it still requires some sort of reference pattern to determine the supportive force. These patterns are mostly based on pre-recorded trajectories from unimpaired volunteers. The major limitation of these patterns is that they are not publicly available. Additionally, most patterns are recorded at a limited number of speeds, while the progress of the patients’ preferred walking speed can be as small as 0.1 km/h.

In this paper, we extend the support strategy that we currently use in out gait trainer LOPES [31]. Within this strategy, patients are supported based on the execution of their gait subtasks, rather than their complete leg movement. Recent simulation and experimental studies [32,33] showed that the muscle activity during walking can be decomposed in different “modules.” Each of these modules can be associated with a specific subtask of walking (e.g., body weight support, forward propulsion or foot clearance). In stroke survivors, each of these subtasks can be impaired to some degree without automatically affecting others. Selectively supporting these subtasks, based on the capabilities and progress of the patient, can be seen as an extension of the “assist-as-needed” principle. Also, the subtasks of both legs can be regarded separately, since in most stroke survivors the paretic leg will be more affected than the non-paretic leg. Controlling gait subtasks, rather than joint angles, also implies that compensatory strategies, like hip circumduction to create more foot clearance, can still be used. Imposing a symmetrical joint-angular reference pattern also limits the possibility of the non-paretic leg to compensate for the deficiencies of the paretic leg.

For the foot-clearance subtask, we developed a controller based on the Virtual Model Control framework [34]. This kind of control provides an elegant way to prevent synchronization problems by only controlling a specific subtask during the corresponding phase of the gait cycle. Using Virtual Models for different subtasks also allows straight-forward adaptation of the support to the subject’s specific needs by only turning on the controllers for impaired subtasks. A pilot study on a small number of healthy subjects already showed that this method allows selective control of foot clearance, while leaving the remaining walking pattern largely unaffected [31]. However, also within a specific subtask, the amount of support needs to match the specific needs of the patient. The support should be such that 1) large errors are prevented, 2) safe walking is guaranteed, 3) small errors and variations over steps are allowed and 4) reliance is minimized. In another pilot study we incorporated the adaptive algorithm, that shaped the impedance as a function of tracking performance, and that was introduced by Emken, et al. [27]. With that pilot study we showed that the stiffness profile converged to a subject-specific pattern, that varied over the gait cycle and matched the subject’s needs [35]. During the various pilot experiments, we also experienced that visual feedback, based on basic gait parameters like foot clearance, is easier to interpret for patients and therapists than feedback in terms of joint angles or interaction torques.

The main contribution of this paper is to show the effectiveness of selective-subtask-support, in conjunction with adaptive support levels, in stroke survivors. Young healthy subjects will be used as a control group. Secondly, a new method to quantify reliance will be tested. Since reliance, or “slacking,” is known to be present in upper-limb robotic support [36], and is considered to be an undesired effect, we try to investigate this phenomena using catch trials. Catch trials are often used in motor learning experiments to evaluate human behavior during prolonged exposure to external stimuli. To our knowledge, this type of methodology, to quantify reliance in lower-limb robotic gait training for stroke survivors, has not been used before. Because reliance is closely related to the feedback that the patient receives, we also developed a system to provide the patient with visual feedback about his performance. Thirdly, we will investigate the use of compensatory strategies in the robotic gait trainer. Since LOPES [28] allows hip abduction, patients are allowed to employ their compensatory strategies in the device. This puts us in the unique position to evaluate whether patients reduce their compensatory strategies when they receive robotic support. Before testing the VMC framework, we will also present a new method, and results, of constructing reference trajectories for the ankle movement at different speeds. First, the pattern is parameterized by defining different key events (minima, maxima etc.), which are extracted from the individual patterns. Next, the walking speed and body-height dependency of the parameters are determined by regression models. These regression models can be used to reconstruct patient-specific ankle movement patterns at any speed.

## Methods

### Reference patterns

#### Subjects

Eleven healthy elderly subjects (five male, six female, age 57.3 ± 5.9, weight 74.9 kg ± 11.9, length 1.70 m ± 0.11) volunteered to participate in an experiment that was setup to collect the reference patterns that are required for VMC of the step height. All subjects had no symptoms of orthopedic or neurological disorders and gave informed consent before participating in the experiments.

#### Experimental protocol

Gait kinematics were recorded using an optical tracking system (Vicon Oxford Metrics, Oxford, UK) at a frequency of 120 Hz. To track the motion of the subject, twenty-one passive reflective markers were attached to bony landmarks on the legs and trunk. The subjects were asked to walk on a treadmill at seven different speeds: 0.5, 1, 1.5, 2, 3, 4 and 5 km/h. After a general familiarization period of three minutes, the subjects walked for three more minutes at each selected speed. During each trial, the subjects did not receive any specific instructions about how to walk on the treadmill. After each trial, the subject had a one-minute break.

#### Data analysis

Different steps were taken to derive the regression models.

### Kinematics

Only the last minute of each trial was used for data analysis. The recorded marker positions were processed using custom-written MATLAB software [37]. Since the proposed VMC approach is end-point based, we do not require the hip and knee angular reference patterns, but only the ankle pattern in Cartesian space.

### Key events and predictor variables

**Figure 1:**
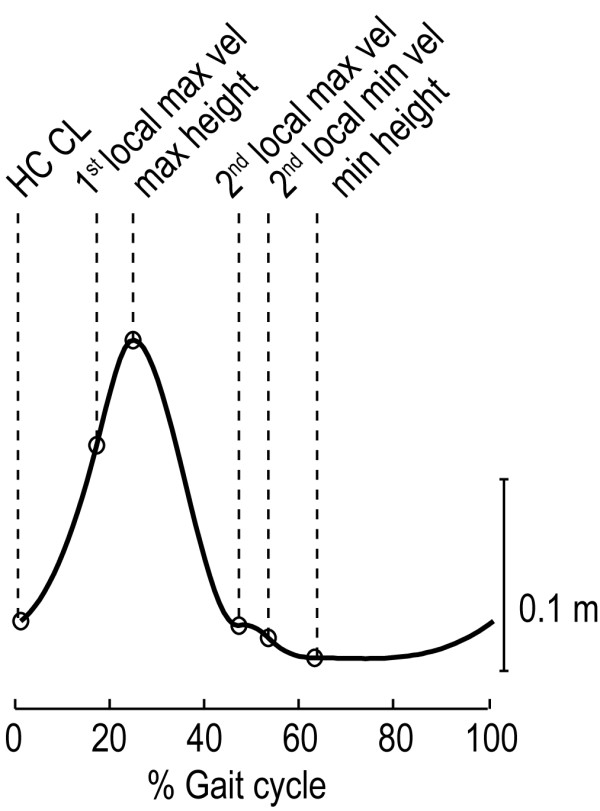
**Selection of key events for the reference ankle-height pattern.** The key events are a selection of extreme values in position and velocity. HC CL represents the key event that is located at Heel Contact of the Contralateral Leg.

The kinematic data was split up into individual strides of the right ankle, based on a phase-detection method developed by Zeni, et al. [38], that used the local maxima in the anterior-posterior position of the heel marker. Each individual stride was parameterized by defining points that corresponded to key events in the gait cycle. For the vertical ankle position, from now on referred to as “ankle height,” these key events included the ankle height at the heel-contact of the contralateral leg (start of the double stance), and a selection of extreme values in position and velocity data. Each key event was parameterized by an index, representing the percentage of the gait cycle at which the key event occurred, and its position and velocity. The median index, position and velocity of the key events were computed for each subject at each walking speed. Figure 1 shows the selected key events for the reference ankle-height pattern.

### Predictor variables

The median index, position, and velocity of the key events were used to construct the regression models. These regression models require a set of predictor variables. We used the following regression formula.

(1) $$y=\beta_{0}+\beta_{1}v+\beta_{2}v^{2}+\beta_{3}l$$

Where *v* represents the walking speed and *l* the body height. *y* represents the index, position or velocity of a particular key event. Stepwise regression [39] was used to test the statistical significance of the predictor variables, using entrance/exit tolerances of 0.05 on the p-values.

### Regression coefficients

**Figure 2:**
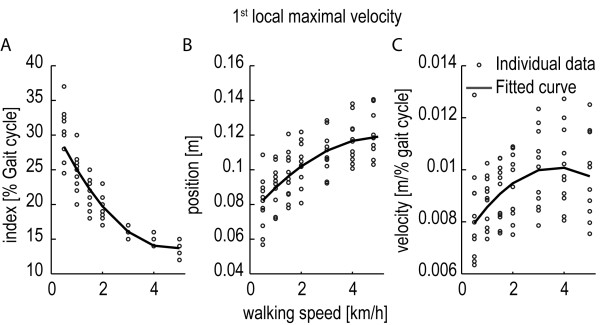
**Relation between walking speed and the index, position, and velocity of a particular key event.** The figure shows the index **(A)**, position **(B)**, and velocity **(C)** of the “1st local maximum velocity” key event at different walking speeds. Each circle represents the median value at a specified walking speed for one subject. The timing of the key event (the index) decreases at higher walking speeds, whereas the position and velocity of the key event increase. The solid line represents the fitted regression model. Stepwise regression showed that the velocity **(C)** of this key event is also dependent on the body height. Here the fitted regression model for the average body height (1,7 m) is shown.

After selecting the appropriate predictor variables for each regression model, robust regression [40] is used to retrieve the final set of regression coefficients (β_x_). Robust regression is an iterative linear regression procedure that uses a tuning function to downweight observations with large residuals. Figure 2 shows an example of how the index, position, and velocity of one key event changes for different walking speeds. The relative position of the key event (the index) decreases at higher walking speeds, whereas the position and velocity of the key event increase. It also shows that these effects are nonlinear. Therefore, the regression models for the index, position, and velocity of this particular key event contain coefficients for the walking speed and walking speed squared. The stepwise regression showed that the body height has no significant contribution to the predictability of the index and position of the key event, whereas for the velocity of this key event it did contribute to the predictability. This indicates that some of the variability in the third figure could be attributed to differences in body height. The lists with the actual values for β_0_, β_1_, β_2_,and β_3_ can be found in the results section.

### Spline fitting

Now that the regression models for the key events are known, a reference pattern can be reconstructed for each walking speed (in the range of 0.5-5.0 km/h). First, the index, position, and velocity of the key events, for a certain speed and body height, are calculated. Next, a cubic spline is fitted between every pair of consecutive key events, resulting in 6 (3rd order) polynomials describing the ankle-height pattern. By definition the position and velocity of the first key event (at 0 percent of the gait cycle) and the end of the last spline (at 100 percent) are equal. The resulting set of splines are merged to construct the reference pattern over the complete gait cycle.

### Validation

To determine the accurateness of the spline-fitting procedure, we compared the constructed splines (based on data from the right ankle of all subjects) with the left ankle pattern of each subject. First, we reconstructed the reference patterns for the set of walking speeds (0.5-5 km/h) for each subject, taking into account the subject’s individual body height. Next, their average left ankle pattern, during the last minute of walking at the different speeds, was calculated and the Root Mean Square Error (RMSE) between both signals was calculated. The resulting RMSE was averaged across subjects, for each speed.

Additionally, the correlation coefficient was used to quantify the similarity between the left ankle pattern and the reconstructed pattern. Both comparisons were performed for the ankle-height profile and the ankle-height velocity profile.

### Selective support of subtasks

#### Subjects

Six elderly stroke survivors (five male, one female, age 57.8 ± 6.4, weight 88 kg ± 12.2, length 1.81 m ± 0.05) volunteered to participate in an experiment that was setup to validate the VMC for the step height. Table 1 lists the clinical description of the stroke survivors in more detail. As a control group, twelve healthy young subjects (six male, six female, age 25.8 ± 2.2, weight 70.3 kg ± 10.9, length 1.77 m ± 0.10) also volunteered to participate in the experiments. All healthy subjects had no symptoms of orthopedic or neurological disorders. Both groups gave informed consent before participating in the experiments.

**Table 1:** Clinical description of patient group

**Subject**	**Age (years)**	**Gender**	**Weight (kg)**	**Length (m)**	**Time since stroke (month)**	**Type of stroke**	**Paretic side**	**FAC**	**DE**	**TBT**	**Proprioception (P/NP)**	**MI leg**
A1	57	male	89.2	1.79	26	Infarction	left	4	1	4	8/8	14
A2	69	female	71.6	1.74	30	Haemorrhage	right	4	2	4	8/8	33
A3	50	male	96.4	1.89	5.5	Infarction	left	5	2	5	8/8	66
A4	59	male	105.5	1.78	72	Haemorrhage	left	4	1	3	7/8	42
A8	54	male	87.1	1.81	12	Infarction	right	4	3	3	8/8	33
A9	58	male	78.0	1.85	7	Infarction	left	5	3	5	8/8	83

FAC = Functional ambulation categories (max = 5).

DE = Duncan Ely test (min = 0, indicating normal tonus, max = 4, indicating rigidity).

TBT = Timed balance test (max = 5).

Proprioception = Outcome of lower extremity proprioception part of Nottingham sensory assessment, paretic (P) vs. non-paretic (NP) (max = 8).

MI leg = Motricity Index score, of the lower extremity (max = 99).

#### Experimental apparatus and recordings

**Figure 3:**
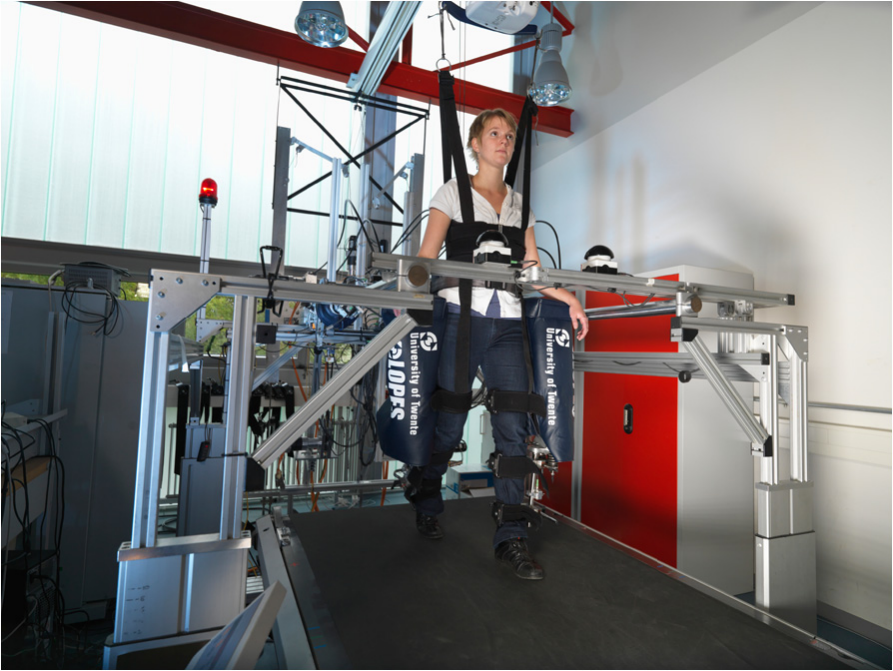
The prototype of the gait rehabilitation robot LOPES.

For the VMC experiments, the prototype of the gait rehabilitation robot LOPES was used (see Figure 3). The system is comprised of a bilateral exoskeleton-type rehabilitation robot above an instrumented treadmill. It is lightweight and actuated by Bowden-cable-driven series-elastic actuators [28]. The robot is impedance controlled, which implies that the actuators are used as torque sources [41]. The exoskeleton offers a freely translatable (3D) pelvis, where the sideways and forward/backward motion is actuated. Furthermore, it contains two actuated rotation axes in the hip joints and one at the knee (abduction/adduction of the hip and flexion/extension of hip and knee). A more detailed description of the exoskeleton design is provided in [28].

Linear and rotary potentiometers measured translations and angular rotations of all degrees of freedom. Kinematics were used to detect heel contact (HC) and toe-off (TO) events. HC and TO were used as triggers to switch the robotic support on and off, and used to segment the data into individual strides.

xPC Target was used for real-time control at 1000 Hz. From the measured exoskeleton joint angles, and the human segment lengths, the ankle position is calculated at each instant of time. Data is collected on the target computer in real-time and then transferred to a host machine, where it was sampled at 100 Hz and stored for off-line analysis, using custom software (MATLAB, Mathworks Inc., Natick, MA, USA). For all subjects we measured joint kinematics (angles and Cartesian positions), the torques applied by the LOPES, and the gait phases.

#### Virtual model control

**Figure 4:**
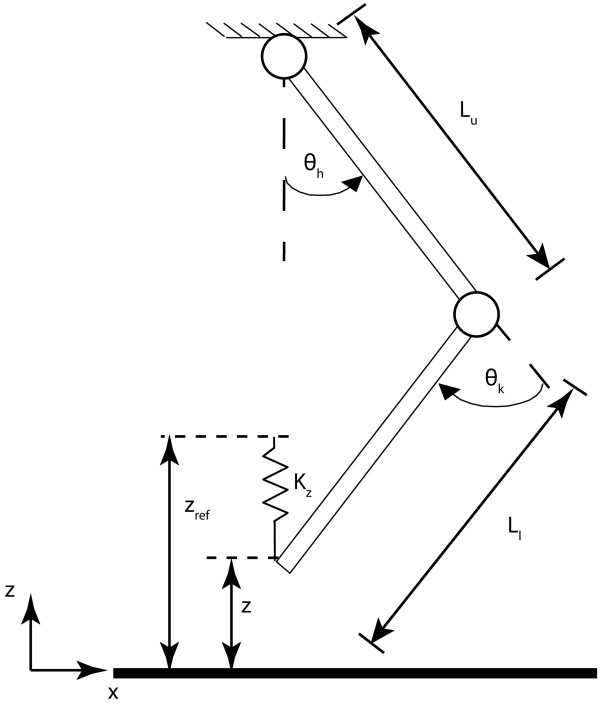
**Schematic representation of the VMC approach.** Z represents the absolute ankle height and Z_ref_ the reference ankle height. L_u_ and L_l_ represent the upper and lower leg length, and ⊖_k_ and ⊖_h_ the knee and hip angle. K_z_ indicates the virtual spring stiffness.

Virtual Model Control was used to selectively support the step-height subtask. The basis of this control method is to define physical interactions with the patient that would assist the gait subtasks. These interactions are then translated into a set of Virtual physical Models (VMs), such as springs and dampers, that can be switched on and off at appropriate times in the gait cycle. The virtual forces, that would be exerted by the VMs, are translated into joint torque commands for the joint actuators. Here we want to support the foot clearance. Therefore, we define a virtual spring (with stiffness K_z_) between the actual ankle height and the reference ankle height (see Figure 4). If the actual ankle height (z) deviates from the reference ankle height (z_ref_), a virtual force (F_z_) is exerted at the ankle, which mimics a therapist lifting the ankle.

(2) $$F_{z}=K_{z}\left(z_{\mathrm{ref}}-z\right)$$

Initial testing showed that no damping was needed, since the human limbs provide a kind of natural damping to the system. The reconstruction of the reference ankle-height pattern is explained in the next section.

The required vertical force is delivered by applying a combination of knee and hip joint torques to the human. The forces of the virtual spring are mapped to joint torques by:

(3) $$\left(\begin{array}{c} \tau_{h}\\ \tau_{k} \end{array}\right){=}_{a}^{h}J^{Th}\left(\begin{array}{c} F_{x}\\ F_{z} \end{array}\right)$$

where *τ* represents the joint torques at the hip and knee, that offers the virtual force in Cartesian coordinates, and _*h*_^*a*^*J*^*T*^ is the transpose of the Jacobian that maps the hip $\left({\dot{\theta }}_{h}\right)$ and knee $\left({\dot{\theta }}_{k}\right)$ angular velocities to the velocities of the ankle in Cartesian coordinates.

(4) $${}_{a}^{h}J=\left[\begin{array}{ll} L_{u}\cos\left(\theta_{h}\right)+L_{l}\cos\left(\theta_{h}-\theta_{k}\right) & -L_{l}\cos\left(\theta_{h}-\theta_{k}\right)\\ L_{u}\sin\left(\theta_{h}\right)+L_{l}\sin\left(\theta_{h}-\theta_{k}\right) & -L_{l}\sin\left(\theta_{h}-\theta_{k}\right) \end{array}\right]$$

For foot-clearance support, only support in vertical direction is required, therefore *F*_*x*_ is zero. The symbols are defined in Figure 4.

#### Reference pattern reconstruction

For the patient group, the reference pattern is reconstructed in the way described before, using the obtained regression models (see spline fitting). In order to investigate the effectiveness of the VMC approach for healthy subjects, we chose to increase the reference pattern, since the subjects were expected to already walk according to the pattern. The shape of the reference pattern is calculated similarly as in the patient group, but the obtained pattern was multiplied such that the maximal ankle height of the reference pattern reached a 15-percent increase with respect to their nominal maximum ankle height. The nominal ankle height of each subject was obtained from a walking trial in LOPES where no support was provided.

#### Synchronization

To prevent synchronization problems a specific subtask is only supported during the phases in which the subtask should be performed. For the step-height support, this indicates that the controller is only active during the double stance (with the contralateral leg in front) and the swing phase. Heel contact and toe-off events were detected in real time based on a phase-detection method developed by Zeni, et al. [38]. To account for alterations in cadence the speed at which the reference trajectory is replayed is scaled to the previous cycle time, and the timer is reset at the contralateral heel contact.

#### Impedance shaping

To adapt the level of support within the step-height subtask, we adopted the error-driven adaptation algorithm of Emken et al. [27]. The algorithm modifies the virtual spring stiffness, at each percentage of the gait cycle, based on the recorded error in the previous steps.

(5) $$K_{z}{}^{i}\left(t\right)=f.K_{z}^{i-1}\left(t\right)+g.\left(z_{\mathrm{ref}}\left(t\right)-z^{i-1}\left(t\right)\right)$$

Where the superscript *i* denotes the i^th^ step cycle, *f* is a forgetting factor set to 0.9, *g* is an error-based gain set to 1800, *K*_*z*_ is the resulting stiffness profile for the ankle height, and t indicates the percentage of the gait cycle, which is estimated based on the previous cycle time. The stiffness was constrained to positive values, since the support is intended to lift the ankle, and not push the ankle downwards, when the ankle is above the reference.

#### Experimental protocol

Before positioning a subject in the LOPES, different anthropometric measurements were taken to adjust the exoskeleton segments lengths. Next, the subject was positioned into the LOPES and the trunk, thigh, and upper- and lower shank were strapped to the exoskeleton.

After a general familiarization period, the preferred walking speed was determined for each stroke survivor individually. During this familiarization period, LOPES was operated in the zero-impedance mode. In this mode the impedance of every joint is set to zero, so the robot provides minimal resistance/assistance to the stroke survivor [42]. All patient trials were performed at the same predefined preferred walking speed. All healthy control subjects walked at 3 km/h.

Next, the stroke survivors, and healthy control subjects, were exposed to selective control of the step height with a compliant (600 N/m), stiff (1200 N/m) and adaptive virtual spring (see impedance shaping). The stiffest of these springs was chosen as having the maximum stiffness that was comfortable for subjects during pilot experiments. For the healthy control subjects all conditions were tested on the right leg only, while the left leg was operated in zero impedance. The stroke survivors were supported on their impaired side.

For the patient group, we intended to use visual feedback to maximally motivate the subjects in taking higher steps. To investigate if subjects are capable of translating the information from a simple visual feedback system into the appropriate action, the visual feedback system was first tested on the healthy control group. The visual feedback system consisted of a screen, showing bars that represented the maximum ankle height of their most recent step. Also, the target height was displayed. Preliminary results showed that healthy subjects were able to use this visual feedback to reach the target height very accurately. Therefore, it was decided to provide this kind of visual feedback to the patient group in almost all conditions.

All conditions were randomized to minimize the effects of fatigue or motor–learning effects. Table 2 lists the different conditions. To evaluate if the robot is influencing the steps without anticipation of the subject, we decided to use catch blocks, where the subject did not receive any support. 7 catch blocks were randomly interspersed among the first 115 steps of support. Each catch block consisted of three steps. Some patients could not walk for 115 consecutive steps because of the severity of their stroke. For these patients the last 10 steps of their trial are discarded, and only fully accomplished catch blocks and exposure blocks are included in the data analysis. To evaluate the effect of prolonged exposure, the trials in the healthy subjects were concluded with 50 steps of continuous exposure.

**Table 2:** List of the tested conditions

	**Stiffness (N/m)**	**Supported leg**	**Visual feedback**	**Abbreviation**
Healthy controls	0	-	off	HZ
	600	right	off	HC
	1200	right	off	HS
	1200	right	on	HSV
	Adaptive	right	off	HA
Stroke survivors	0	-	on	PZV
	600	paretic	on	PCV
	1200	paretic	on	PSV
	Adaptive	paretic	on	PAV
	Adaptive	paretic	off	PA

#### Data analysis

In general, the effectiveness of the step-height controller was assessed by determining how well the set reference values were attained, and how the support affected other aspects of walking. First, the data was segmented into separate steps based on the heel contact events [38]. Next, different spatiotemporal gait parameters were extracted from the ankle trajectories: the maximal ankle height (step height), the step length, and the cycle time. The maximum ankle height is the maximum vertical displacement of the ankle during swing. The step length is the relative horizontal displacement of one ankle with respect to the opposite ankle at the moment of heel contact. All gait parameters were normalized with respect to their nominal values. This allowed for comparison across subjects and conditions. For the healthy subjects, as well as the stroke survivors, the nominal values were recorded during a trial in which they walked in the zero impedance mode. Additionally, the relative duration of the different gait phases was calculated. All parameters were obtained for the exposure, as well as the catch blocks. Group averages were calculated for the stroke survivor and healthy control group.

To investigate the reduction of compensatory strategies, we also determined the maximum knee flexion, maximum hip abduction, and maximum pelvic height for the stroke survivors during the different conditions. Stroke survivors, with stiff-knee gait, for example, often fail to reach enough toe clearance and use different compensatory strategies to overcome their reduced knee flexion. Common strategies are a circumduction strategy, pelvic hiking, and vaulting. Vaulting is caused by an increase of the plantar flexion of the non-paretic leg, pushing the pelvis upward and creating more foot clearance on the paretic size. We hypothesize that, when stroke survivors experience step-height support, they reduce their compensatory strategies. Thus, assisting one subtask might automatically correct gait kinematics elsewhere.

### Statistical analysis

To investigate the selectivity of the VMC support, we first used a one sample t-test to determine whether the percentage change in the spatiotemporal parameters differed from 0 percent. If the step-height support significantly influenced one of the defined spatiotemporal parameters, we used a paired t-test to assess whether there was a statistically significant difference between the conditions with the compliant and stiff virtual spring. All statistical tests were performed with SPSS Statistics (IBM Corporation, Armonk, NY, USA). The level of significance was defined at 5 percent.

## Results

### Regression models for the reference patterns

**Figure 5:**
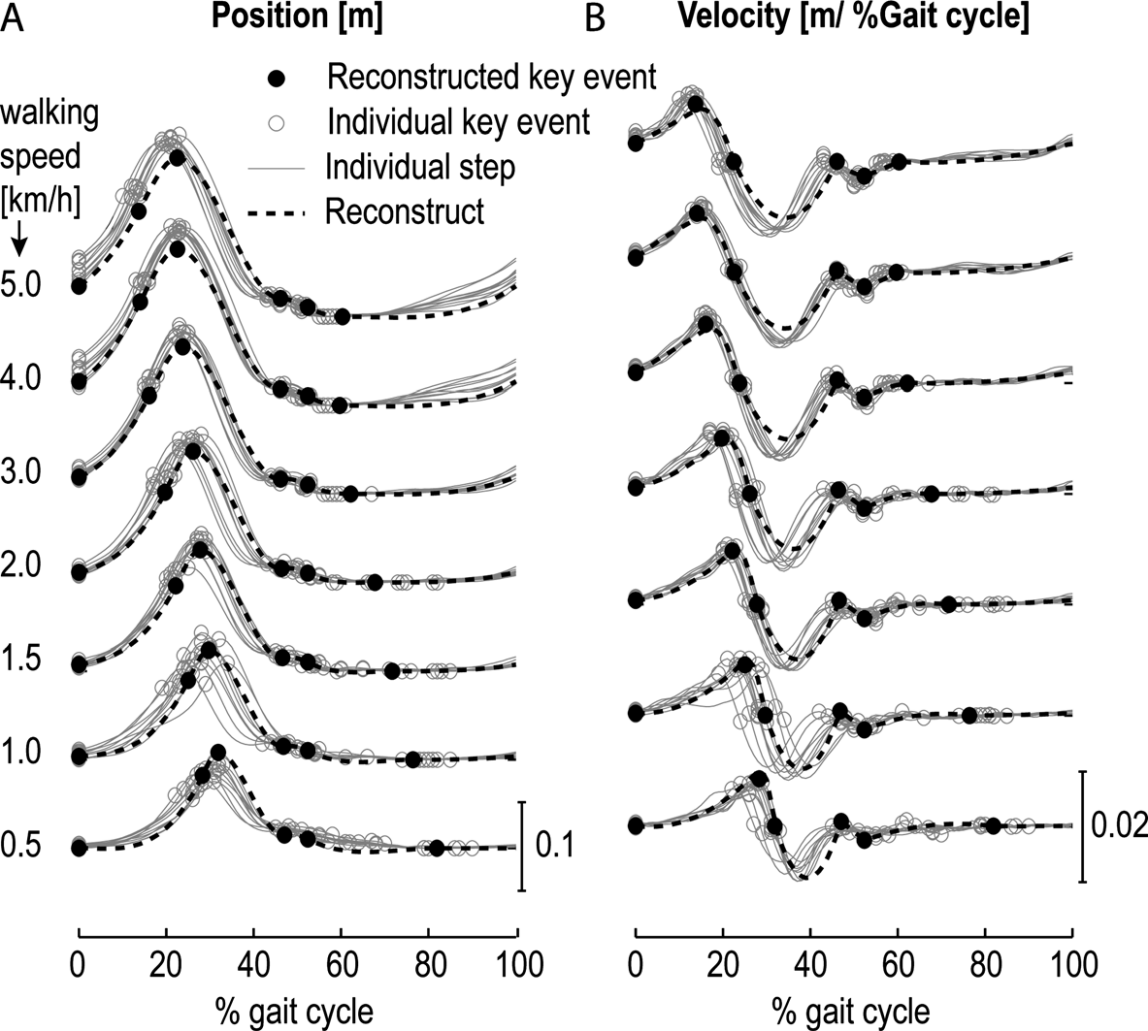
**Typical example of the reconstructed ankle-height patterns.** Graph A shows the individual steps (gray lines), together with the detected key events (gray circles), at different walking speeds, for a specific subject. The black filled circles represent the predicted key events for this particular subject, based on the obtained regression models. The black line represents the spline, which is fitted through the predicted key events. Graph B shows the velocity profile.

The timing, position and velocity of the key events were highly dependent on the walking speed (see Figure 5). Generally, the different subjects showed the same dependencies. However, there was considerable variation between the subjects in the timing, position and velocity of the key events at a specific walking speed (see Figure 2). The stepwise regression, and subsequent robust regression, showed that most key events were linearly and/or quadratically dependent on speed (see Table 3). The body height did not influence the timing (index) of the key events. Of all positions it only influenced the “maximal height” key event and it influenced the velocity at three of the six key events. The Root Mean Square Error (RMSE), in the prediction of the timing of the key events, was <2 percent of the gait cycle (except for the timing of the “minimal height”). The RMSE, in the prediction of the position, was maximally 1.44 cm and was maximally 0.11 cm/%gait cycle for the velocity. Figure 5 also shows that the key events could be predicted well using the regression equations.

**Table 3:** Regression models for the index, position and velocity of the different key events

		**β**_**0 **_**(Intercept)**	**β**_**1 **_**(speed)**	**β **_**2 **_**(speed**^**2**^**)**	**β**_**3 **_**(body height)**	**RMSE**
**Index**	HC CL	0	-	-	-	0
	1^st^ local max vel.	32.0	−7.79	0.826	-	1.92
	max height	34.4	−5.27	0.579	-	1.70
	2^nd^ local max vel.	47.3	−0.609	0.0725	-	1.09
	2^nd^ local min vel.	52.4	-	-	-	1.26
	min height	88.2	−13.3	1.55	-	4.87
		**β**_**0 **_**(Intercept)**	**β**_**1 **_**(speed)**	**β **_**2 **_**(speed**^**2**^**)**	**β**_**3 **_**(body height)**	**RMSE**
**Position**	HC CL	−0.387 x10^-2^	0.768 x10^-2^	-	-	0. 572 x10^-2^
	1^st^ local max vel.	7.41 x10^-2^	1.73 x10^-2^	−0.165 x10^-2^	-	1.36 x10^-2^
	max height	−2.63 x10^-2^	3.57 x10^-2^	−0.362 x10^-2^	6.64 x10^-2^	1.44 x10^-2^
	2^nd^ local max vel.	1.49 x10^-2^	-	0.0221 x10^-2^	-	0. 345 x10^-2^
	2^nd^ local min vel.	1.05 x10^-2^	-	-	-	0. 231 x10^-2^
	min height	0	-	-	-	0
		**β**_**0 **_**(Intercept)**	**β**_**1 **_**(speed)**	**β **_**2 **_**(speed**^**2**^**)**	**β**_**3 **_**(body height)**	**RMSE**
**Velocity**	HC CL	2.79 x10^-3^	0. 732 x10^-3^	-	−1.75 x10^-3^	0. 466 x10^-3^
	1^st^ local max vel.	−6.22 x10^-3^	1.54 x10^-3^	−0. 207 x10^-3^	7.95 x10^-3^	1.08 x10^-3^
	max height	0	-	-	-	0
	2^nd^ local max vel.	−3.31 x10^-3^	-	0.0307 x10^-3^	2.35 x10^-3^	0. 970 x10^-3^
	2^nd^ local min vel.	−2.57 x10^-3^	-	-	-	0. 628 x10^-3^
	min height	0	-	-	-	0

Speed is expressed in km/h and body height in m.

HC CL - Heel contact contralateral leg.

“–“ indicates that that particular predictor variables does not contribute to the predictability of the key event.

**Figure 6:**
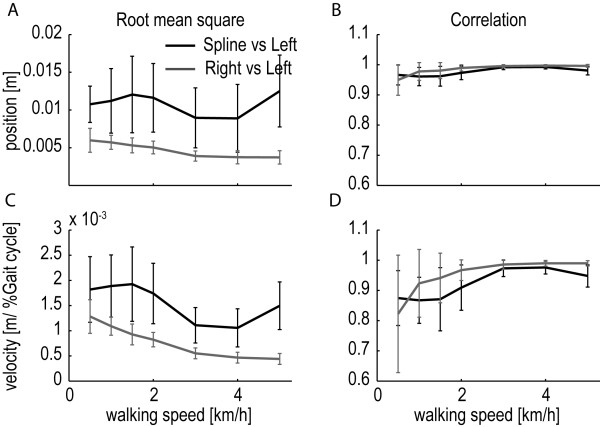
**Validation of the reconstructed ankle-height patterns. A**: RMSE between the left ankle-height pattern and the reconstructed spline (black line), and the RMSE between the left ankle-height pattern and the right ankle-height pattern (gray). Both measures were averaged across subjects for each walking speed. The error bars indicate the standard deviation. **B**: Correlation between the left ankle-height pattern and the reconstructed spline (black line), and the correlation between the left ankle-height pattern and the right ankle-height pattern (gray line).Graph C and D show similar figures for the validation of the velocity profile.

From the predicted key events, a reference ankle-height pattern was reconstructed for every subject and walking speed. We validated these patterns by comparing them with the measured patterns of the left leg (NB the regression equations were fitted on data of the right leg). The reconstructed patterns fitted the measured data well (see Figure 6). The RMSE, averaged across subjects, was around 1 cm for all walking speeds and the average correlation coefficient was larger than 0.95, for the low speeds, and showed even larger values for higher walking speeds. Since the error in predicting the key events is reflected in the reconstructed patterns, these RMSE values were close to the average RMSE in the prediction of the position of the key events (see Table 3, average position RMSE = 0.79 cm). The large correlation coefficients are in line with the small RMSE in the prediction of the timing of the key events. Also, the reconstructed velocity profiles matched the measured velocity profiles well (see Figure 6), though the correlations were a bit lower, especially for the lower velocities.

As a reference, we also calculated the RMSE and correlation coefficient between the measured right and left ankle patterns. These values provide an indication of the achievable fitting quality (see Figure 6). The correlations between the reconstructed spline and left leg data were very close to the correlations between the left and right leg data, whereas the RMSE values were approximately twice as large.

### Healthy control group

#### Selective and gradual support

**Figure 7:**
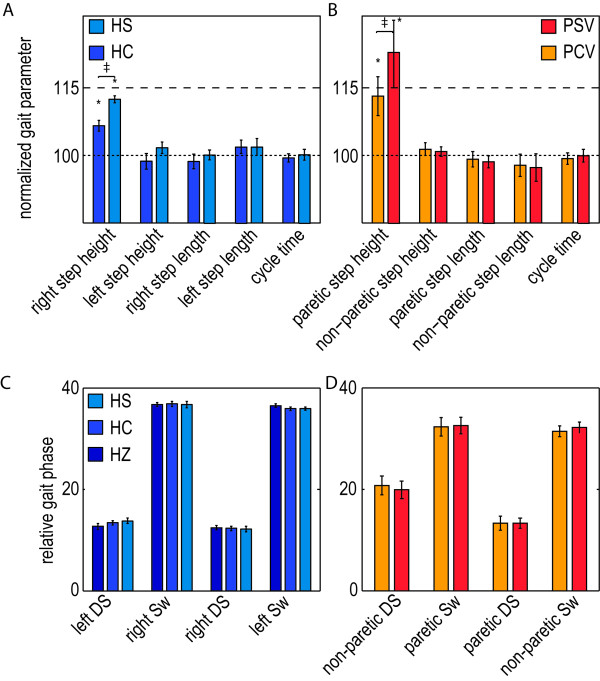
**Selective and gradual foot-clearance support in the healthy control subjects and stroke survivors. A**: Mean normalized gait parameters for the trials in which the healthy subjects walked with the compliant (HC) and stiff (HS) step-height VMC. The mean parameters are calculated during the last 10 steps of the last exposure block. **C**: Mean relative contribution of the different gait phases to the cycle time for both tested conditions. As a reference, also the relative gait phases during walking in the zero impedance mode (HZ) are shown. **B**: Mean normalized gait parameters for the trials in which stroke survivors walked with the compliant (PCV) and stiff (PSV) step-height VMC, in combination the visual feedback. Mean parameters are calculated during the five steps of the longest exposure block. **D**: Mean relative gait phases for both tested conditions. The error bars indicate the standard error of the mean. *p < 0.05. ++ indicates a significant difference between the compliant and stiff VMC.

**Figure 8:**
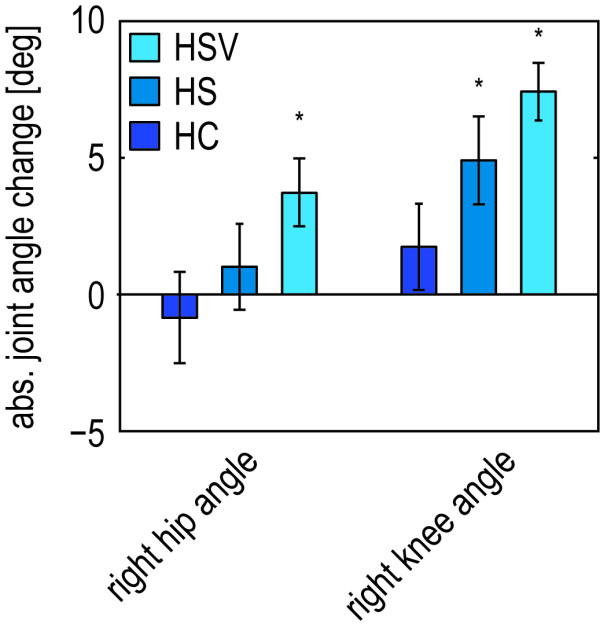
**Increase in hip and knee angle during foot-clearance support for the healthy control subjects.** Mean absolute changes in the hip and knee angle for the trials in which the healthy subjects walked with the compliant step-height VMC (HC), stiff VMC (HS), and with the stiff VMC in combination with the visual feedback (HSV). Then mean parameters are calculated during the last 10 steps of the last exposure block. The error bars indicate the standard error of the mean. *p < 0.05.

One of the goals of this study was to show the feasibility of selectively and gradually supporting step height during gait training. Providing step-height support resulted in a selective support of this specific subtask. It significantly increased the right step height, whereas it did not significantly affect the other basic gait parameters, like the left step height, step length, cycle time, or the relative duration of the different gait phases (see Figure 7). Analyzing the gait kinematics showed that the increase in step height was primarily caused by an increase in knee flexion. For the stiff controller, the average knee angle increased with 4.9 degrees at the moment of maximum ankle height, whereas the hip angle at that moment increased with only 1 degree (see Figure 8). The average maximum joint torques, that causes these changes, were 10 Nm hip extension and 9.6 Nm knee flexion. The support was also gradual, since the use of the stiff controller resulted in a significant increase in step height compared to the compliant controller.

#### Non-adaptive support does not induce reliance

**Figure 9:**
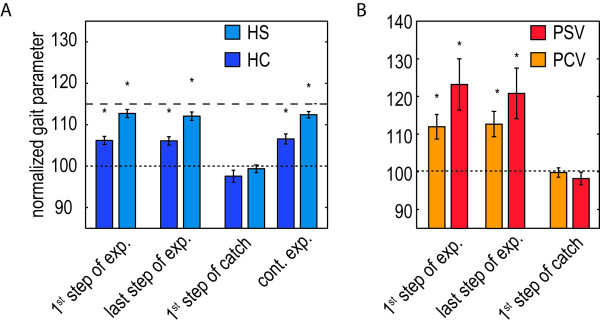
**Effect of non-adaptive support on reliance. A**: Mean normalized step height during different steps of the trial in which the healthy subjects walked with the compliant (HC) and stiff (HS) step-height VMC. Mean parameters are calculated during the first step of the exposure block, during the last step of the exposure block, during the first step of the catch block and during continuous exposure. Continuous exposure is based on the last 10 steps of the last exposure block. **B**: Mean normalized step height during different steps of the trial in which the stroke survivors walked with the compliant (PCV) and stiff (PSV) step-height VMC, in combination with visual feedback. Mean parameters are calculated during the first step of the exposure block, during the last step of the exposure block, and the first step in the catch block. The last exposure block, with 50 steps of continuous exposure, was not included in the protocol of the stroke survivors. The error bars indicate the standard error of the mean. *p < 0.05.

We did not find any evidence for reliance of the subjects on the provided support, when they are exposed to continuous non-adaptive support. No significant difference between the initial exposure (first steps of the exposure blocks) and prolonged exposure (last steps of the exposure block) was found (see Figure 9). The step height during the first step of the catch block also revealed no signs of reliance. It shows that the subjects drop back to their baseline, without any significant undershoot, which was to be expected when reliance would occur (see Figure 9). This holds for the compliant as well as the stiff controller. Even when the subjects received continuous support for a longer period of time (50 steps of continuous exposure), the step height did not significantly differ from the initial exposure.

#### Visual feedback enhances performance and active participation

**Figure 10:**
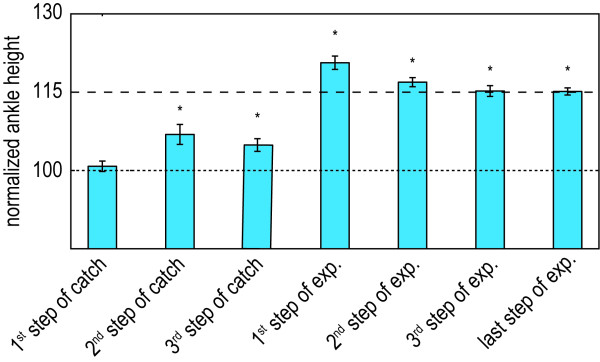
**Influence of adding visual feedback to the support.** Mean normalized step height during different steps of the trial in which the healthy subjects walked with the stiff VMC in combination with the visual feedback (HSV). The step height is calculated during the first, second, and third step of the catch block, during the first, second, and third step of the exposure block, and during the last step of the exposure block. The error bars indicate the standard error of the mean. *p < 0.05.

With visual feedback, the healthy subjects reached an average increase of 14.5 percent in step height during continuous exposure, while the reference was set to 15 percent. This demonstrates that they can easily translate the simple information displayed on the screen into the appropriate hip and knee angular response. The visual feedback resulted in an additional increase in hip and knee flexion compared to the stiff controller (see Figure 8). The results also demonstrate that the subjects use the feedback to actively increase their step height within one step after the support has switched off (see Figure 10). After the support is switched on, unexpectedly, they receive additional support, which creates an overshoot. The subjects easily adapt to the additional support and reach the target value again within two steps.

### Stroke survivors

#### Selective and gradual support

Providing step-height support to the stroke survivors resulted in a selective increase in step height, without significantly affecting the other basic gait parameters, including the relative duration of the different gait phases (see Figure 7). The support was also gradual, since the use of the stiff controller resulted in a significant increase in step height compared to the compliant controller. The stroke survivors show a larger standard error of the mean of the paretic step height, compared to the right step height of the control group. For the compliant controller the increase in nominal step height ranged between 0 and 26 percent, whereas the increase in step height due to the stiff controller ranged between 0 and 44 Percent. For the stiff controller the average maximum joint torques were 21.0 Nm hip extension and 17.4 Nm knee flexion. The stroke survivors also show an asymmetry in stance phase (see Figure 7), which is often observed in stroke survivors [43].

#### Impedance shaping

**Figure 11:**
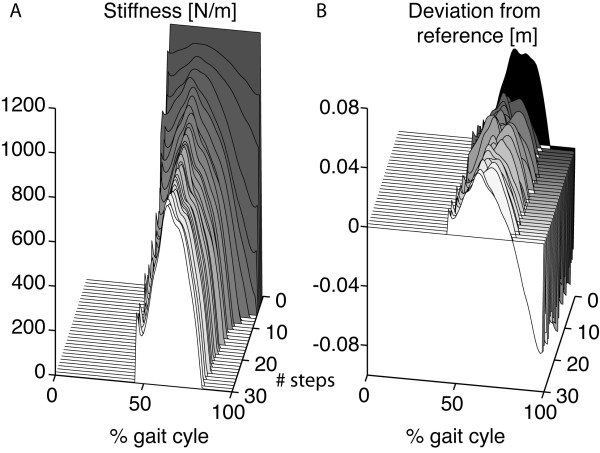
**Typical example of the working principle of the impedance-shaping algorithm in a stroke survivor.** Graph **A** demonstrates how the stiffness shapes from a constant stiffness of 1200 N/m to a personal stiffness profile after around 30 steps for stroke survivor A3. Note that the step-height VMC is only active during the double stance, with the non-paretic leg in front, and the paretic swing phase (approximately 50–100 percent of the gait cycle), and that the stiffness has a lower limit of 0. Graph **B** shows the course of the deviation from the reference pattern over multiple steps. The black area shows the error before the controller is switched on.

The experiments showed that the impedance-shaping algorithm was effective in adapting the amount of support to the stroke survivor’s individual capabilities on a step-by-step basis (see Figure 11). Starting from the initial stiffness (1200 N/m), the adaptive algorithm causes a gradual increase in stiffness where a kinematic error persists, and a clear reduction in stiffness where the ankle is above the reference (i.e., a negative deviation from the reference in Figure 11B). After ± 30 steps, the stiffness profile reached a steady state, where the forgetting factor and the deviation of the ankle from the reference pattern are in equilibrium. Figure 11B demonstrates that the stiffness can be greatly reduced without automatically compromising the overall kinematic error. That is, the difference between initial stiffness (1200 N/m) and final stiffness is much clearer than the change in kinematic error.

**Figure 12:**
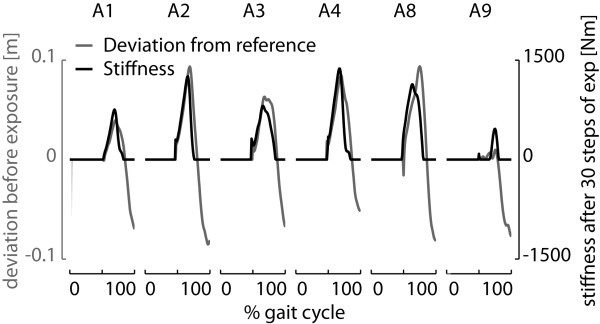
**Shape of the stiffness profile after convergence.** The figure shows the initial deviation of the ankle from the reference trajectory (gray) together with the converged stiffness profile (black). For all patients, the stiffness shaped according to the initial deviation from the reference.

With the impedance-shaping algorithm, the spring stiffness was shaped such that it reflected the initial deviation of the ankle from the reference trajectory for all patients (see Figure 12). Although the stiffness converged to a personal profile for each patient, the highest stiffness occurred at the start of the swing phase for all patients.

#### Non-adaptive support does not induce reliance

Similar to the healthy control group, we did not find any evidence that indicated that the stroke survivors started to rely on the support. We did not find a significant difference between the initial exposure and the end of the exposure blocks (see Figure 10). This is also confirmed by the catch blocks, which show that during support (with the compliant or stiff VMC) the stroke survivors drop back to their baseline, without any significant undershot.

#### Visual feedback enhances performance and active participation

**Figure 13:**
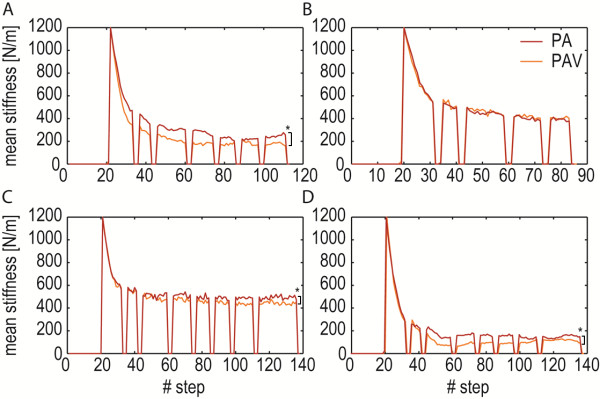
**Adding visual feedback to adaptive support.** Mean stiffness over the course of the walking trial in which four stroke survivors (A1 (**A**), A4 (**B**), A8 (**C**), and A9 (**D**)) walked with the impedance-shaping algorithm with (PAV) and without visual feedback (PA). In three patients, the mean stiffness over the last five steps of the last exposure block was significantly lower when the patients received visual feedback. This indicates that 1) these patients improved their performance with the help of the visual feedback and 2) the impedance-shaping algorithm lowered the impedance when the patients improved their performance. *p < 0.05.

To evaluate if stroke survivors can utilize the visual feedback, we compared the trials where the stroke survivors received adaptive support combined with visual feedback (PAV), with the trials where the stroke survivors received adaptive support only (PA). For this comparison, data from only four patients was available, since the PA condition could not be tested on two patients due to fatigue. In three of the four patients, the mean stiffness over the last five steps of the last exposure block was significantly lower when the patients received visual feedback (see Figure 13). This indicates that 1) these patients improved their performance with the help of the visual feedback and 2) the impedance-shaping algorithm lowered the impedance when the patients improved their performance.

#### No reduction of compensatory strategies

**Figure 14:**
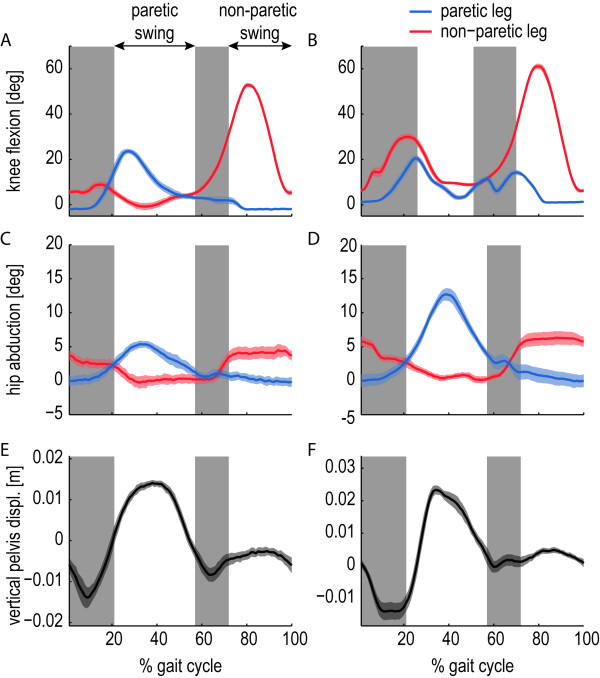
**Two typical examples of gait adaptations seen in stroke survivors.** Both patients (A4 (left) and A1 (right)) show one or more compensatory strategies to overcome a reduced foot clearance due to stiff-knee gait (**A** and **B**). They show an increase in hip abduction (**C** and **D**), and an increased pelvic height during the paretic swing phase, compared to the non-paretic swing phase (**E** and **F**).

During the experiments, the stroke survivors showed different combinations, and degrees, of compensatory strategies to overcome their reduced knee flexion. All patients showed a larger paretic hip abduction range (hip circumduction) and an increased pelvic height during the paretic swing phase (vaulting). Figure 14 shows two typical examples of stroke survivors with stiff-knee gait, who use a vaulting strategy and/or a hip circumduction strategy. None of the patients reduced their compensatory strategies during the assistance. Although the use of the stiff controller resulted in an average increase of 8.8 degrees in the maximum paretic knee flexion, and all patients reported that they felt the assistance in their paretic leg, we did not find a significant reduction in the hip abduction of the paretic leg, or a decrease in pelvic height during the paretic swing phase.

## Discussion

The purpose of this study was to assess the effectiveness of selectively supporting the step height during the swing phase. First, we derived regression models for the key events of the reference ankle-height pattern. These models can be used to reconstruct patient-specific reference patterns at any speed. The proposed step-height VMC was tested on healthy subjects and chronic stroke survivors, and proved effective in selectively influencing the step height. Additionally, the step height could be manipulated easily by changing the impedance levels. Incorporation of an impedance-shaping algorithm resulted in an adaptation of the impedance to the specific needs of every individual stroke survivor. Catch trials were used to investigate whether healthy subjects, or stroke survivors, would start to rely on the robotic support, but revealed no signs of reliance. The step height parameter was used to provide intuitive visual feedback. Both groups were able to utilize this feedback. We did not find evidence that the stroke survivors reduced their compensatory strategies when support was provided.

### Reference pattern reconstruction

A large part of this paper concerns the reconstruction of the reference patterns. Throughout the literature, different strategies exist to determine these reference patterns. Most reference patterns are based on pre-recorded trajectories from unimpaired volunteers walking on a treadmill [24,29,44,45], or based on walking in the device while it is operated in a transparent mode [21,27], or with the motors removed [46]. Patient-specific patterns can be obtained by recording the gait trajectories while the patient walks with manual assistance [21,27], or by defining joint patterns based on movements of the unimpaired limb [47]. Most methodologies, however, have certain considerations that limit the use of the recorded trajectories to a specific application.

A major limitation of most of these reference patterns is that it is unknown how to correct for changes in speed. Most pre-recorded trajectories are recorded at a limited number of speeds, while the progress of the patients’ preferred walking speed can be as small as 0.1 km/h. The coupling between the right and left leg [47] will change at different speeds and the recorded pattern, obtained during manual assistance [21,27], will only be valid for that specific speed. Scaling algorithms can be used to compensate for changes in speed or cadence [48]. Most scaling algorithms, however, apply scaling in time, amplitude and offset, whereas also the (relative) timing of the maximum joint angles changes at different speeds.

For the patterns recorded in the gait trainer itself, another limitation should be noted. Due to the mass and inertia of the device, and/or imperfections of the transparent mode, these patterns might not match with the ones recorded during free walking. Emken, et al., found that the added inertia resulted in a slightly higher stepping pattern compared to free walking [22], while others found a significant and relevant decrease in knee angular range due to the device [42].

Most pre-recorded trajectories are obtained by rescaling the gait pattern to a percentage of the gait cycle, and taking the mean across subjects. This introduces another issue. Averaging normalized data can result in an underestimation of the extremes in the gait pattern, when the subjects have a different distribution of the gait events throughout the gait cycle [49].

Therefore, we developed a method where the gait pattern is parameterized by defining different key events (minima, maxima etc.), which all have a timing, position, and velocity. Next, the walking speed and body-height dependency of the parameters are determined by regression models. This way, the extreme value in the reconstructed pattern is actually based on the extreme values of the individual patterns, even when the extremes occur at another percentage in the gait cycle.

Another advantage of the proposed method, compared to other available methods, is that it can be used to construct a reference ankle-height pattern at each particular walking speed between 0.5 and 5.0 km/h, for persons with different body heights. This allows the physical therapist to easily increase the training speed, even within a single walking session. Speed-dependent reference pattern adjustments are also essential when the patient is in control of the walking speed, either manually or with intuitive speed-adaptation algorithms [50].

The proposed method is also generally applicable and can be applied to reconstruct speed-dependent reference patterns for joint angles. Different studies have already shown that peak joint kinematics are dependent in a linear and/or quadratic way on walking speed [51], and that its occurrence (timing) is also speed dependent [52].

There were some limitations in deriving the regression equations, which are related to the relatively low number of subjects (11) in this study. Due to this small number, we did not derive separate equations for male and female subjects, whereas systematic effects of gender of kinematics have been reported [53]. The range of body heights in this group of subjects was limited (1.52 m to 1.86). However, this range is expected to be sufficient for the majority of the elderly population.

### Selective and gradual support

The results from the stroke survivors and healthy control group showed that the step-height VMC could selectively influence the step height. Supporting the step height did not significantly affect other spatiotemporal gait parameters, like non-supported step height, step length, or relative gait phase duration. Although the subjects were free to adapt their cadence, no change in cycle time was observed. As expected, the support was also gradual, a higher stiffness resulted in a closer approximation of the target values.

On average, the stroke survivors received more supportive hip and knee torque. At baseline, the stroke survivors walked more below the reference than the healthy subjects, resulting in more supportive torque.

The stroke survivors also showed a larger standard error of the mean of the paretic step height, compared to the right step height of the control group. For the healthy subjects, the reference ankle-height pattern was scaled such that it reached a 15-percent increase with respect to their nominal maximum ankle height. For the stroke survivors, the reference pattern was purely based on the regression formulas. The stroke survivors who were less affected, and almost reached their target value without support, showed a smaller (relative) increase in step height, compared to the patients that performed less without the support.

### Impedance shaping

Selective-subtask-support already allowed us to focus the robotic support on the subtasks that are impaired. However, also within a subtask, the amount of support needs to be minimized to the personal needs of the patient. Aoyagi, et al., already suggested that by scheduling the impedance as a function of the gait cycle, the assistance can be further personalized [21]. This, however, is impossible for the operator to manually adjust. Therefore, we chose to adopt an adaptive algorithm, that shaped the impedance based on the tracking performance, that was suggested by Emken, et al. [27].

Emken, et al., reported that the impedance converged repeatedly over separate trials [27]. Although we only performed one trial with the adaptive algorithm per patient, the impedance profile shaped according to the initial error between ankle and the reference trajectory for all patients. This indicates that the shaped impedance was directly related to the patient’s incapabilities.

All stroke survivors converged to a stiffness profile where the stiffness was highest at swing initiation. This is in agreement with the trials where a constant stiffness was used. There, most of the assistive torques were exerted during swing initiation, indicating that that phase requires most of the support. Because of the provided torques during initial swing, the leg was propelled upward with a higher velocity, and required less support during the remainder of the swing phase. Anderson, et al., already demonstrated the importance of knee angular velocity at swing initiation in normal gait. They showed that the knee angular velocity at heel off was the main determinant for the maximal knee angle, and foot clearance, during swing [54]. Reduced angular velocity, and foot clearance, in stiff-knee gait is suggested to be caused by an abnormal knee flexion during swing initiation. Kerrigan, et al., and Riley, et al., found an inappropriate activity in at least one of the quadriceps muscles during the pre-swing or initial swing phase [55,56], which inhibit a normal knee flexion. Kerrigan, et al. [55], also reported that patients with delayed heel rise achieved less peak knee flexion. The patients included in this study also showed a delayed heel rise. So, providing support during this phase seems like a natural, and the most effective, way to increase the maximum knee angle, and subsequently foot clearance.

Apart from shaping the impedance to the patient’s individual needs, minimizing the impedance also allows more variability within the stepping pattern, which has been shown to promote motor learning in mice [18]. Emken, et al., reported an increase in variability in maximum step height and step length, but could not verify whether increasing the variability during gait training had a positive effect on EMG activity levels [27]. In our study, we did not investigate the variability within the gait pattern. We did see a clear reduction in the impedance levels where the stroke survivors required less support, which allows them to vary their steps in a more natural way compared to walking with a stiff controller. The possibility to make small gait variation was also promoted by using a unidirectional spring that only provided support in taking a higher step, thus not constraining the ankle when it reached above the reference.

### Reliance

Based on previous pilot experiments [35], and computational models of movement training [57], we hypothesized that the stroke survivors and healthy controls would start to rely on the support, such that when assistance is no longer provided, their performance becomes worse. Previous studies, that let subjects adapt to external force fields, already showed that the human motor system can be modeled as a process that greedily minimizes a cost function, consisting of a weighted sum of kinematic error and effort [58,59]. In these studies, a forgetting factor is introduced in the human effort, which models that the human continuously tries to accomplish the prescribed movement with reduced effort.

In this study, however, we did not find patients, or healthy subjects, who started to rely on the support. A likely explanation for the patient group could be the visual feedback, which we did not use in the pilot experiment [35]. The visual feedback provided them with information about their performance on a step-by-step basis, increasing their motivation and reducing the changes of reliance.

Also in the healthy control group, who did not receive visual feedback in most trials, we did not observe reliance. To evaluate the effects of prolonged exposure, the trials were concluded with 50 steps of continuous exposure. This block might have been too short for the subjects to explore the benefits of the support and start to rely on it. The relatively low impedance levels might contribute to this effect.

Also the task instruction and type of support might explain our findings. In most motor learning experiments, a disturbing force field is applied and the subjects are asked to reduce the error. To reduce the error, the subjects have to produce additional effort to overcome the disturbance. During this process, they continuously try to minimize the trade-off between reducing their effort and increasing the error [58,59]. In our experiments, the subjects experience a force field that decreases, rather than increases, the performance error. Here, the subjects are not challenged to provide additional effort, which might not elicit them to reduce their effort.

The fact that a relative small movement error can cause the subjects to trip might also have contributed to the fact that these subjects did not start to rely on the support. This would indicate that the weight of the error in the cost function increases compared to the reduction in effort. Bays, et al., already suggested that humans can change the weighting of different costs, according to the task and type of the movement [60].

Although the chances that reliance will occur are reduced by minimizing and localizing the support with the impedance shaping algorithm, two issues remain. First, the algorithm cannot distinguish between a decrease in effort due to reliance or due to fatigue. In both cases, the algorithm will increase its support. Second, subjects might still, consciously or unconsciously, reduce their effort over time and consequently receive more support. Emken, et al., showed that, to effectively assist-as-needed, the robot must reduce its assistance at a rate that is faster than that of the learning human [59]. They stated that reliance can be prevented by setting the forgetting factor to a lower value than the learning rate of the subjects. They also state that determining the learning rate for neurological patients can be difficult because of their impaired motor control due to spasticity, muscle weakness, and synergies. Therefore, we chose to set the forgetting factor based on a stable convergence of the stiffness pattern within approximately 30 steps.

Finally, one might argue that to eliminate reliance one should apply resistive forces rather than supportive forces. In fact, error-enhancing therapy is suggested to be more effective than assistive therapy [20,61]. For some training exercises, where movement errors do not impose serious safety issues, this might be true. For robotic treadmill training, where small movement errors can have large consequences, this strategy may be inappropriate.

### Visual feedback

In this study, very simple visual feedback was provided in the form of the step-height parameter. We showed that both the stroke survivors and the healthy controls were capable of utilizing this information effectively. Providing visual feedback to the healthy controls led to a very close approximation of the reference values. Adding visual feedback to the trials, in which the stroke survivors received adaptive support, led to lower impedance levels in three of the four patients, indicating that these patients are additionally motivated by the visual feedback.

The key element of any form of feedback is that it displays the subject’s effort in an intuitive manner. Different forms of feedback are available. A review performed by Teasell, et al. [62], concluded that there is a positive effect of EMG feedback in patients after stroke. Others use the subject’s kinematics to display their performance [25,29], or the interaction force between user and robot, like in the Lokomat [63].

A disadvantage of the latter approach is that it is only applicable to position-controlled gait trainers. In these type of gait trainers, the additional effort of the subject is reflected on the screen, but is not reflected in their gait pattern. This might decrease the motivation of the subject. Thus, to optimize visual feedback, the gait trainer needs to be compliant. In more recent versions of the Lokomat, Duschau-Wicke, et al., introduced a more patient-cooperative strategy, effectively making the robot more compliant [25]. In their study, they used body kinematics as visual feedback.

To optimize the feedback, factors like the amount of information and its frequency need to be investigated. Also the complexity of the feedback is important - do we need detailed information from every joint, or combined information from several joints, like the ankle position? Banala, et al. [29], only displayed the ankle position in the sagittal plane. Our results suggest that only showing the maximum ankle height of the last step is already sufficient to control the hip and knee joint such that the subject takes a higher step. Also, for the therapist himself, we expect that feedback in the form of basic gait parameters will be easier to interpret, compared to joint angles or ankle trajectories.

The primary goal of visual feedback is, of course, to contribute to the long-term changes in relearned gait kinematics. Kim, et al. [26], used the ALEX to induce gait modification in healthy adults. They reported that a combination of visual and force guidance resulted in larger modifications in step height that maintained longer, persisting up to two hours, whereas only visual guidance or only force guidance evoked changes that did not last beyond the 10-min retention test. Although we did not investigate retention, our experience with visual feedback is encouraging, and can serve as a starting point in the investigation about how to optimize gait training in such a way that short-term gait adaptation can become long-lasting gait modification.

### Compensatory strategies

The VMC approach, used in this study, is an end-point-based-control strategy. This implies that within a certain subtask there is more freedom to walk, or choose a certain strategy. For example, different patients might choose different strategies to accomplish appropriate foot clearance. With the step-height VMC, the patients are left free in the strategy they use to clear their foot and will only receive support when this task is not executed successfully. This means that compensatory strategies [64,65], like pelvic hiking, hip circumduction, or vaulting [66,67], which are seen in most stroke survivors, can still be employed. Joint control limits the use of these strategies. Additionally, imposing a symmetrical joint-angle pattern limits the possibility of the non-paretic leg to compensate for the deficiencies of the paretic leg. Although these compensatory strategies do not contribute to a more symmetric walking pattern, they do increase basic gait function [68,69]. Some even advocate teaching compensatory strategies because of time and financial limitations [70]. Thus, because it is still largely debated whether the focus of robotic gait training should be on restitution of a normal walking pattern or on these compensatory strategies, they should not be overruled.

The use of these compensatory strategies might even become redundant when support is provided on the impaired subtask that evokes these compensatory strategies. We hypothesized that providing support on one subtask, i.e. foot clearance, would reduce the need for the patient to employ his compensatory strategies. Although all our stroke survivors showed compensatory strategies without support, none of them reduced their compensatory strategies with support. During the experiments, the stroke survivors received no specific instructions about how to walk on the treadmill. Therefore, they might not have been triggered to reduce their compensatory strategies. Also, the limited time that the stroke survivors walked in the LOPES during the experiments, in combination with the amount of time it would take to un-teach their adapted strategies, might be a reason for the unchanged kinematics. In the future, we might even develop special VMC modules that suppress compensatory strategies to promote restitution of a symmetrical walking pattern.

### Related work

Different support methods have been suggested to correct the gait pattern of neurological patients. However, none of the compliant, or interactive, support methods has been evaluated in large-scale clinical trials. To guide potential clinical trials, the differences between our and other approaches will be explained. The method presented in this paper can be best compared to the “virtual tunnel” approach. Banala, et al., implemented this virtual tunnel approach, which was previously described by Chai, et al., [30], and trained two chronic stroke survivors with the ALEX [29]. Their tunnel consisted of a healthy-control template and the assistance was composed of a normal force, that simulated the virtual walls, and a tangential force that helped the ankle move along the trajectory. A similar virtual tunnel strategy is implemented in the Lokomat to train iSCI patients. Duschau-Wicke, et al., also implemented a “moving window” that limits free movement to a region of the tunnel, similar to the tangential force in the ALEX [25]. In contrast to Banala, et al., they defined a torque field in joint space rather than a force field in Cartesian space. There are three main differences between the above-mentioned control strategies and the control strategy presented in this study.

First, both the ALEX and the Lokomat use some sort of support that potentially helps the ankle, or joint, move along the trajectory. The tangential force, used by Banala, et al. [29], decreases when the ankle deviates from the trajectory, thus the ankle is only pushed along the path when the ankle is close to the desired trajectory. Duschau-Wicke, et al. [25] use the moving window, that is synchronized with the user’s cadence [71], to assist the user. In our study, no tangential force, or moving window, is used. Within the subtask-support strategy, step timing and foot clearance are two separate subtasks. Here, we only supported foot clearance. This allowed the subjects to freely change their timing, if they wished to do so. Still, subjects did not adapt their timing. For bilateral affected iSCI patients, who experience difficulties during swing initiation, or gait initiation in general, gait-timing assistance might be useful. In that case an additional VMC in the horizontal plane can be added. Our experience with stroke survivors suggests that the non-paretic leg can take care of the gait timing and the paretic leg will follow.

Second, both studies use a virtual tunnel that lifts the ankle [29], or increases joint angles [25], but can also do the opposite when the subject performs above the reference. In this study, a unidirectional spring was used, because the support is intended to support the subject in taking higher steps, and not push the ankle downwards, when the ankle is above the reference.

Third, in contrast to the Lokomat, the support of subtasks is an end-point-based-control strategy, rather than a joint-angle-based-control strategy. As mentioned before, joint-angle-based-control strategies exclude the use of compensatory strategies.

### Future applications of selective support

The key goal of future research is to expand the concept of subtask support. Support in taking higher steps is an important part of the rehabilitation process, but other subtasks might also require assistance. A new VMC, that assists patients in taking more symmetric steps, is currently under development, and its interaction with other subtask controllers is being investigated.

For severely affected patients, body weight support systems (BWSS) are often used. Alternatively, VMC can also be used to partially support the body weight by attaching a vertical virtual spring to the hips. In that case, the forces, required to bear your own body weight, are provided in terms of hip and knee torques, rather than lifting the body externally. This allows normal sensory input from the foot soles, which is essential in order to generate natural gait kinematics [72,73]. VMC for body weight support also allows easy modulation of the amount of support between the different legs, since stroke patients primarily need support during the stance phase of the affected leg. It also enables separate control of body weight support and balance support, which can be considered as two separate subtasks, either of which can be impaired to a certain degree. BWSS, with an overhead harness, not only provide a force in the pure vertical direction, but also in the horizontal plane that stabilizes the body. Pilot experiments have shown the feasibility of body weight support with VMC [74]. The possibilities of VMC for balance support are now being investigated.

Finally, we started preliminary tests with an intuitive speed-adaptation algorithm, in which the patient can move freely over the treadmill and the speed is automatically adapted when the patient deviates from the center of the treadmill. In conjunction with the obtained speed-dependent reference patterns, this will provide the therapist and patient with tools to easily adapt the treadmill speed to the capabilities and progress of the patient, without the need to manually change the control settings.

## Conclusion

In this study we implemented, and evaluated, a VMC strategy for selective and gradual support of gait subtasks. Here we focused on one specific subtask, i.e. increasing foot clearance. Initially, we derived and provided regression models that can be used to reconstruct patient-specific ankle movement patterns based on body height and walking speed. The RMSE between the predicted and actual trajectory was around 1 cm for all walking speeds. The proposed method can also be applied to reconstruct speed-dependent reference patterns for joint angles. Experiments with healthy subjects, and chronic stroke survivors, showed that with the proposed VMC approach, the step height could be selectively and gradually influenced, without affecting other spatiotemporal gait variables. In conjunction, we tested an impedance-shaping algorithm, which shaped the impedance to the patient’s individual needs. The provided support did not result in reliance on the support for both the stroke survivors as well as the healthy control groups. Providing visual feedback to the user resulted in an increased active contribution in all healthy subjects and three of the four stroke survivors. The presented VMC approach, and impedance shaping, can be crucial for the development of new rehabilitation strategies and robotic gait trainers. It allows automatic localization and minimization of the support, which increases active patient contribution and promotes functional recovery.

## Competing interests

The authors declare that they have no competing interests.

## Authors’ contributions

BK and EA carried out the experiments, collected and processed the data, and wrote the manuscript. HK participated in the design of the study and contributed to the revision of the manuscript. All authors read and approved the final manuscript.
